# Modeled associations between university physical education mechanisms and students’ mental health

**DOI:** 10.3389/fpsyg.2026.1829590

**Published:** 2026-09-23

**Authors:** Ran Zhu, Zheng Ma, Min Yan, Galiya Madiyeva, Yessenova Kamchat

**Affiliations:** 1 Faculty of Philosophy and Political Science, Al-Farabi Kazakh National University, Almaty, Kazakhstan; 2 Faculty of Medicine and Health, Al-Farabi Kazakh National University, Almaty, Kazakhstan; 3 Faculty of Physical Education, Huanggang Normal University, Huanggang, Hubei, China; 4 Faculty of Martial Arts, Shandong Sport University, Jinan, China

**Keywords:** Huber loss, mental health, moderate exercise, physical education, sparrow search

## Abstract

Mental health issues among students are becoming increasingly serious, and physical exercise is closely linked to both physical and mental health. Therefore, there is a correlation between physical education and students’ physical and mental well-being. When examining the relationship between physical education mechanisms in higher education institutions and students’ mental health, fluctuations such as individual differences often appear in the data. To address this issue, this paper proposes the “Sparrow Search Tree Network” (SSTNet), a model that employs the “Sparrow Search Algorithm” (SSA) to systematically analyze the relationship between the two. Additionally, SSTNet incorporates the Huber loss function to enhance the model’s stability when dealing with outlier samples. The results indicate that SSTNet achieves higher predictive accuracy, model stability, and generalization capability than the control model. Applying SSTNet to analyze the modeled associations between university physical education mechanisms and students’ mental health indicates that moderate exercise intensity coupled with a high-quality teaching and interactive environment is associated with lower DASS-21 total scores, that is, with lower psychological distress rather than with better scores on a distress scale.

## 1 Introduction

Recently, under the dual pressure of academic burden and employment opportunities, many university students have become mentally ill, suffering from ailments such as anxiety, depressive disorder, and emotional dysregulation (Seguí-Odriozola, 2025; Han S. S.et al., 2025). These psychological problems may affect not only students’ attention to and performance at school but also their personality development, social adjustment, and even their physical and mental wellbeing in the long term. Improving the quality of higher education researching student development has become more pressing, as many mental health issues remain almost unfixed in higher education. It is not only the universities and colleges themselves that must improve their psychological counseling and intervention service systems (Han et al., 2025a), but they also have increasingly accepted that physical education is the foundation, backbone, and sustaining force of students’ mental wellbeing (Han et al., 2025a; Xu et al., 2025). Physical education reduces students’ psychological burden, improves their emotional state, and enhances psychological resilience (Kohl and Cook, 2013; Qiu et al., 2025). University physical education provides a practical setting in which associations between physical education mechanisms and students’ mental health can be examined by modifying course type, exercise content, and the student-teacher interaction (Han et al., 2025b).

Most current research uses cross-sectional questionnaire surveys combined with correlation analysis or linear regression models to examine the association between physical exercise and mental health (Du et al., 2025; Zhu et al., 2024). These methods are commonly used to describe linear associations between variables in a general way (Blanca et al., 2018; Huang et al., 2025).

However, physical education mechanisms and mental health outcomes have multidimensional characteristics, highly non-linear relationships, and complicated interactions (Cong et al., 2025). Under these conditions, conventional linear models have well-recognized limitations, which make it difficult for researchers to identify the modeled associations between physical education mechanisms and student mental health.

Moreover, mental health data also suffer from high subjectivity (i.e., high variance between individual subjects), large fluctuation ranges, and frequent outliers or measurement bias in the sample. Extreme Gradient Boosting (XGBoost) is a scalable tree-boosting algorithm originally proposed by Chen and Guestrin. It combines a regularized learning objective with an additive ensemble of classification and regression trees and is widely used for high-dimensional and non-linear mapping relationships (Asselman et al., 2023; Chen, 2020). Graph-based deep learning models have also been used for high-dimensional representation learning (Chen, 2024). We find that the outcome of the model is highly dependent on the correct selection of loss functions and effective hyperparameter settings.

The Huber loss function, originally proposed by Huber (1964), is a robust loss function that combines the advantages of mean squared error (MSE) with mean absolute error (MAE) (Wang et al., 2022; Terven et al., 2025). For small prediction errors, it behaves like squared loss and provides a smooth gradient for efficient model training; for large errors, it behaves like absolute loss and prevents outliers from exerting excessive influence on the optimization process (Wang et al., 2022). This is particularly relevant for mental health questionnaire data, which may contain individual heterogeneity, response noise, and abnormal observations. Recent machine-learning studies have emphasized that appropriate loss-function selection is crucial for improving robustness and predictive stability (Terven et al., 2025).

The Sparrow Search Algorithm (SSA), originally proposed by Xue and Shen (2020) and Xue and Shen (2024), is a swarm-intelligence optimization algorithm inspired by the foraging and anti-predation behavior of sparrows, with parameter optimization explored in subsequent studies (Willmott and Matsuura, 2005; Chai and Draxler, 2014; Nagelkerke, 1991; Cheng et al., 2024). In SSA, individuals in the population are divided into discoverers, joiners, and sentinels, which jointly balance global exploration and local exploitation during the search process. Recent studies have shown that SSA and its improved variants have been widely applied to complex optimization problems and machine-learning model optimization because of their simple structure, flexible search mechanism, and good global search capability (Nayak et al., 2023; Awadallah et al., 2023). In the present study, SSA is used to optimize key parameters of the XGBoost-based model, such as tree depth, learning rate, sampling parameters, regularization coefficients, and the Huber loss parameter, thereby reducing the uncertainty and inefficiency associated with manual parameter selection.

This study develops and validates SSTNet, a robust machine-learning framework that integrates SSA-based hyperparameter optimization, XGBoost modeling, and Huber loss. The primary objective of this study is methodological: to improve the predictive accuracy, robustness, and generalization capability of mental health prediction using noisy and heterogeneous student data. The substantive analysis of university physical education mechanisms and students’ mental health serves as the empirical application and validation context of the proposed model. Based on this objective, the study addresses the following research questions:

RQ1: Can the proposed SSTNet model achieve better predictive accuracy, robustness, and generalization than commonly used baseline models when analyzing student mental health data?
RQ2: Does the integration of SSA-based hyperparameter optimization and Huber loss improve the performance and stability of the XGBoost-based prediction framework?
RQ3: Based on the SSTNet model, which university physical education mechanism factors, including physical education class participation frequency, exercise intensity adaptation, and teaching interaction quality, are most strongly associated with students’ mental health outcomes?

Accordingly, the following hypotheses are proposed:

*H1*: SSTNet will outperform baseline machine-learning models in predicting students’ mental health outcomes, as indicated by lower MAE and RMSE values and higher R^2^ values.

*H2*: The integration of SSA-based hyperparameter optimization and Huber loss will improve the robustness and predictive performance of the XGBoost-based framework.

*H3*: Exercise intensity adaptation and teaching interaction quality will show stronger associations with students’ mental health outcomes, specifically stronger associations with lower DASS-21 total scores (lower psychological distress), than physical education class participation frequency.

In the hypotheses above, mean absolute error (MAE) is the average absolute deviation between predicted and observed values (Terven et al., 2025; Willmott and Matsuura, 2005). Root mean square error (RMSE) is the square root of the mean squared prediction error and is more sensitive to large residuals than MAE (Terven et al., 2025; Chai and Draxler, 2014). The coefficient of determination (R^2^) measures the proportion of variance in the dependent variable explained by the model; higher R^2^ values indicate better goodness of fit (Terven et al., 2025; Nagelkerke, 1991). Accordingly, lower MAE and RMSE values and higher R^2^ values indicate better predictive performance. Students’ mental health outcomes were operationalized as DASS-21 total scores. The 21-item Depression Anxiety Stress Scales (DASS-21) assess depression, anxiety, and stress; item scores are summed within each subscale, multiplied by two, and then combined into a total score ranging from 0 to 126. Higher DASS-21 total scores indicate greater overall psychological distress not better mental health (Lovibond and Lovibond, 1995; Henry and Crawford, 2005; Cao et al., 2023; Chen et al., 2023; Zhang Z. et al., 2024).

There are three innovations in this paper.

1. This study proposes the framework, Sparrow Search Tree Network (SSTNet), aiming to utilize SSA for optimizing key model parameters. This approach replaces the time-consuming and costly manual selection and grid search methods traditionally employed in model and grid design processes, thereby achieving superior model outcomes.
2. The Huber loss function employs quadratic loss for minor errors and absolute loss for significant errors, effectively mitigating the impact of outliers and enhancing model robustness. Integrating the Huber loss function into SSTNet improves the model’s adaptability to noisy data and outliers, thereby boosting prediction accuracy and generalization performance.
3. Employing the SSTNet model for data analysis significantly mitigates the interference of anomalous samples and noise within mental health datasets, thereby reducing the detrimental impact of abnormal training samples on predictive outcomes. This renders the new model more stable when applied to authentic university education data environments.

The primary objective of this study is to develop and validate SSTNet, a robust machine-learning framework for analyzing noisy and heterogeneous student mental health data. SSTNet integrates SSA-based hyperparameter optimization, XGBoost modeling, and Huber loss to improve prediction accuracy, robustness, and generalization. The substantive analysis of university physical education mechanisms and students’ mental health serves as the empirical application and validation context of the proposed model. In this integrated framework, SSTNet is used not only to predict students’ mental health outcomes but also to identify the relative importance of key physical education mechanism factors, including physical education class participation frequency, exercise intensity adaptation, and teaching interaction quality.

## 2 Method

SSTNet consists of XGBoost (Asselman et al., 2023; Chen, 2020), the Sparrow Search Algorithm (Xue and Shen, 2020; Cheng et al., 2024), and Huber loss (Huber, 1964; Wang et al., 2022), leading to the establishment of a very stable analytical model with very high predictive performance. To illustrate the structure of the proposed model, Figure 1 presents the SSTNet framework, including the SSA-based optimization module, the XGBoost prediction module, and the Huber loss function used to improve robustness.

**Figure 1:**
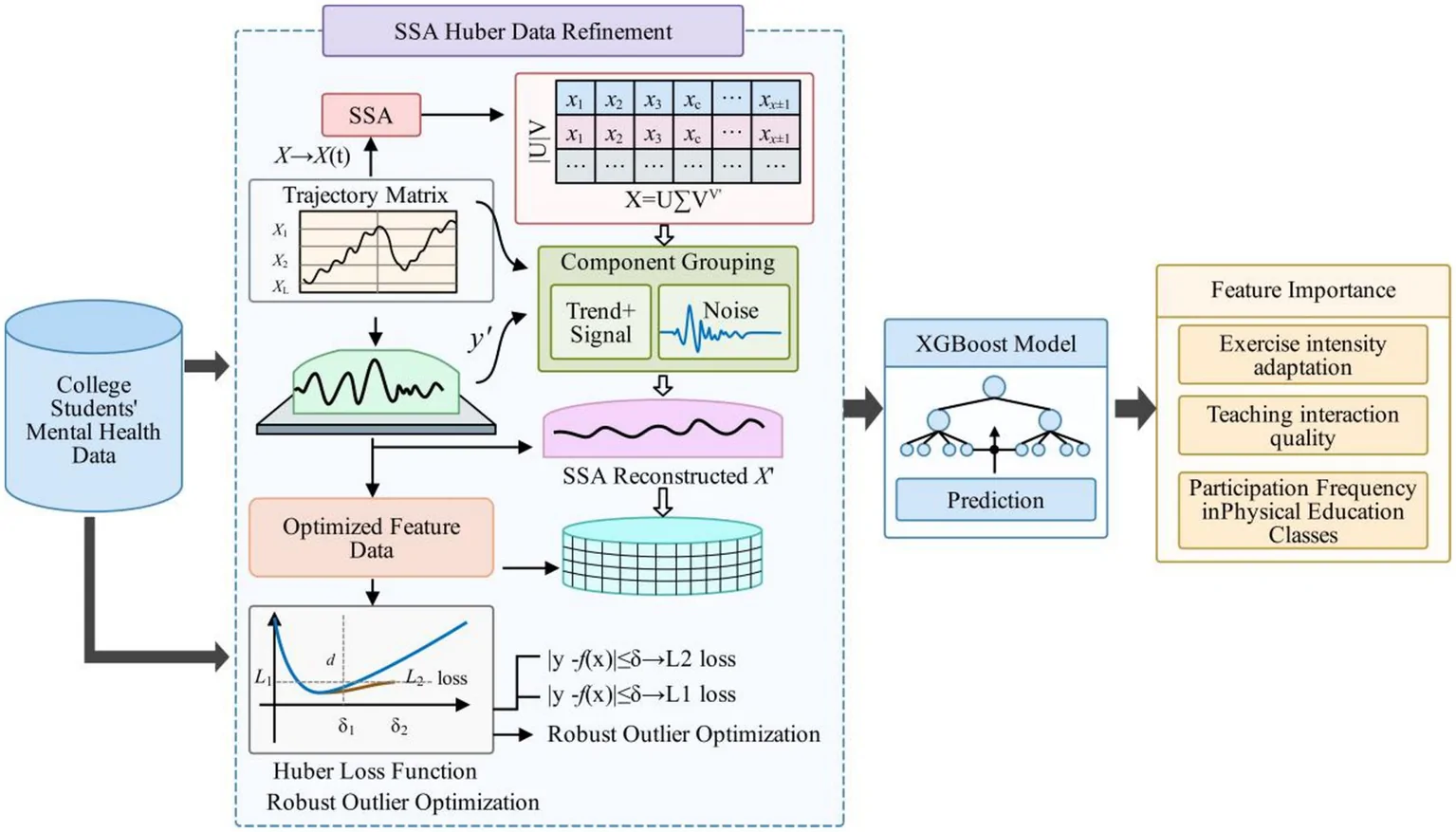
Structure of the SSTNet model. Source: Author-created.

### 2.1 The SSTNet of the overall structure

The SSTNet model primarily comprises SSA, XGBoost, and the Huber loss function. SSA simulates sparrow foraging and vigilance behaviors to perform global optimization of key model parameters, thereby enhancing model performance (Xue and Shen, 2020; Xue and Shen, 2024). XGBoost, serving as the core predictive model, integrates multiple decision trees through gradient boosting, enabling effective handling of complex nonlinear relationships and improving predictive accuracy (Asselman et al., 2023; Chen, 2020). The Huber loss function combines the advantages of squared loss and absolute loss, mitigating the impact of outliers on model training while enhancing robustness and generalization capability (Huber, 1964; Wang et al., 2022). This tripartite integration elevates the SSTNet model’s predictive accuracy and stability.

### 2.2 The SSA and XGBoost structure

The SSA algorithm calculates parameters by simulating the behavior of sparrows searching for food and fleeing from predators. The algorithm has fast convergence and excellent stability (Cheng et al., 2024; Xue and Shen, 2024). It allows explorers to traverse the solution space actively and exchange information about solutions, and joiners can use the information from explorers to continue the local search. In the SSA algorithm, the space occupied by each individual in the population represents a potential good solution. Specifically, if a population with *N* sparrow in D-dimensional search space is to be optimized, the state of the population is as Equation 1.

$$A=[\begin{array}{cc} a_{1}^{1} & a_{1}^{2}\cdots a_{1}^{d}\\ \begin{array}{c} a_{2}^{1}\\ \vdots \\ a_{n}^{1} \end{array} & \begin{array}{c} a_{2}^{2}\\ \vdots \\ a_{n}^{2} \end{array}\;\begin{array}{c} \cdots \\ \ddots \\ \cdots \end{array}\;\begin{array}{c} a_{2}^{d}\\ \vdots \\ a_{n}^{d} \end{array} \end{array}]$$ (1)

where *d* denotes the dimension of the search space, and *n* denotes the population size. The fitness value for the entire population is as shown in Equation 2.

$$F_{a}=[\begin{array}{c} f([\begin{array}{ccc} a_{1}^{1} & a_{1}^{2} & \begin{array}{cc} \cdots & a_{1}^{d} \end{array} \end{array}])\\ f([\begin{array}{ccc} a_{2}^{1} & a_{2}^{2} & \begin{array}{cc} \cdots & a_{2}^{d} \end{array} \end{array}])\\ \cdots f([\begin{array}{ccc} a_{n}^{1} & a_{n}^{2} & \begin{array}{cc} \cdots & a_{n}^{d} \end{array} \end{array}]) \end{array}]$$ (2)

where each row’s value in $F_{a}$ represents the fitness value of an individual. Within the SSA algorithm, discoverers possess priority exploration rights; they not only locate high-quality solution regions first but also guide joiners in search directions. Their position updates are as shown in Equation 3.

$$a_{\mathrm{id}}^{t+1}=\{\begin{array}{c} a_{\mathrm{id}}^{t}\cdot \exp(\frac{-i}{\alpha \cdot T}),R_{2}<\mathrm{ST}\\ a_{\mathrm{id}}^{t}+Q\cdot L,R_{2}\geq \mathrm{ST} \end{array}$$ (3)

where *t* denotes the current iteration count, *T* represents the maximum iteration count, and α is the perturbation factor control coefficient, a random variable uniformly distributed between [0, 1]. $a_{\mathrm{id}}^{t}$ denotes the position information in the d-th dimension for the i-th individual at iteration t. $a_{\mathrm{id}}^{t+1}$ denotes the position information in the d-th dimension at iteration *t* + 1; *Q* denotes a random number following a standard normal distribution; L represents the unit vector; R₂ is the alert threshold, a random number between [0, 1]. ST is the safety threshold, a random number between [0.5, 1].

The follower adopts a competitive trailing strategy, optimizing its position by continuously monitoring the food source discovered by the leader. Its position update is as shown in Equation 4.

$$a_{\mathrm{id}}^{t+1}=\{\begin{array}{c} Q\cdot \exp(\frac{aw_{d}^{t}-a_{\mathrm{id}}^{t}}{i^{2}}),i>\frac{n}{2}\\ ab_{d}^{t+1}+|a_{\mathrm{id}}^{t}-ab_{d}^{t+1}|A^{+}\cdot L,\text{otherwise} \end{array}$$ (4)

where $aw_{d}^{t}$ denotes the globally worst position, $a_{\mathrm{id}}^{t}$ represents the optimal position occupied by the participant, and $a_{\mathrm{id}}^{t+1}$ indicates the position of the individual with the highest fitness in the t + 1th iteration. A is a 1 × *d* matrix whose elements are either random or −1, satisfying $B^{+}=(B^{T}){\left(BB^{T}\right)}^{-1}$ .

The initial positions of sentinels employ a random initialization strategy, with position updates as shown in Equation 5.

$$a_{\mathrm{id}}^{t+1}=\{\begin{array}{c} ab_{d}^{t}+\beta (a_{\mathrm{id}}^{t}-ab_{d}^{t}),f_{i}\neq f_{g}\\ a_{\mathrm{id}}^{t}+K_{v}(\frac{a_{\mathrm{id}}^{t}-aw_{d}^{t}}{|f_{i}-f_{w}|+\varepsilon }),f_{i}=f_{g} \end{array}$$ (5)

where *β* is the step size control parameter, a random number following a normal distribution with mean 0 and variance 1. Kv is a random number between [−1, 1]. $f_{i}$ , $f_{w}$ , and $f_{g}$ denote the current sparrow fitness, worst fitness, and best fitness, respectively. ε is a sufficiently small number to prevent the denominator from becoming zero.

The advantage of XGBoost is, by using optimization methods of regularization function terms and weighted quantitude sampling, the training efficiency that improves the accuracy of the predictive effect is maximized. In the same way, many weak classifiers are merged together and the structure risk of the forecast was minimized in the algorithm into order to obtain a strong predictive model to achieve a well-developed predictive model to become a broadly applicable analytical effect in all fields (Chen, 2020; Sibindi et al., 2023). XGBoost is used to predict the predicted value of all the trees, as shown in Equation 6.

$${\hat{y}}_{i}=\sum_{t=1}^{K}f_{i}(x_{i}),f_{i}\in ℱ$$ (6)

where $f_{k}$ are the K-th tree and ℱ is the set of all possible trees.

The regularization function of SSTNet can be described as shown in Equation 7.

$$\Omega (f)=\mathrm{\gamma T}+\frac{1}{2}\lambda \sum_{j=1}^{T}w_{j}^{2}$$ (7)

where ‘*T*’ is the number of leaf nodes of the tree, γ and λ are the regularization coefficients, and Wj represents the weight of the j-th leaf node. To optimize the objective function, XGBoost adopts a greedy algorithm and a second-order Taylor expansion, choosing the optimal split point at each step to maximize the objective function gain. The information provided by the first and second derivatives of the loss function greatly improves the efficiency of XGBoost’s optimization process.

### 2.3 Huber loss function

When the Huber loss function is applied to XGBoost models, the MSE characteristic is utilized to deal with small errors and provides a smooth gradient, and the MAE characteristic is used in the case of larger errors to avoid excessive penalty (Huber, 1964; Terven et al., 2025). The Huber loss function not only improves the stability of XGBoost when handling outliers but also improves the prediction power of the model (Elharrouss et al., 2025). The Huber loss function is as shown in Equation 8.

$$L_{\delta }(y,f(x))=\{\begin{array}{c} \frac{1}{2}{\left(y-f(x)\right)}^{2},|y-f(x)|\leq \delta \\ \delta |y-f(x)|-\frac{1}{2}\delta^{2},|y-f(x)|>\delta \end{array}$$ (8)

where y is the true value, fx is the predicted value, and δ is the parameter of the Huber loss function. If the absolute value of the error in the prediction does not exceed δ, the loss function utilizes a quadratic term and ensures the gradient is smooth for small errors, whereas if the large error is the case, the loss function utilizes a linear term to avoid excessive sensitivity. When the true value is set to 0 and the predicted value ranges from −10 to 10, the MAE, MSE, and Huber loss (δ = 1) curves are as is shown in Figure 2.

**Figure 2:**
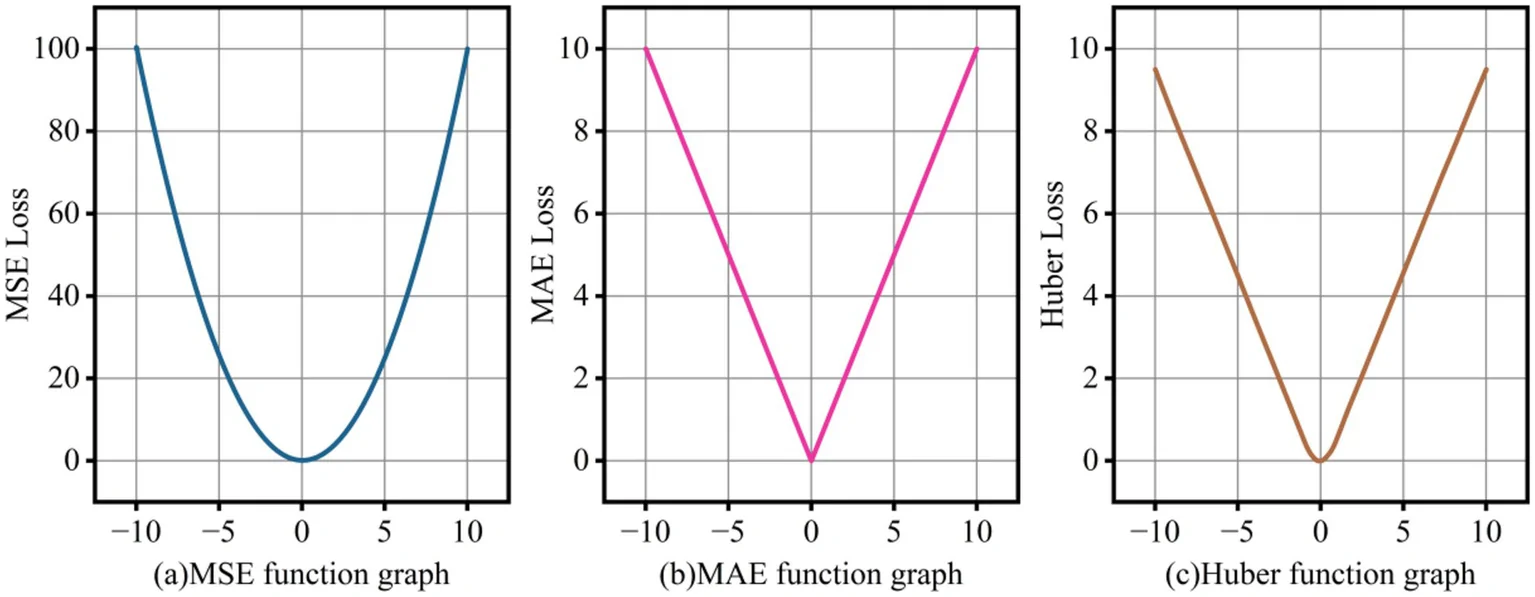
Comparison of MSE, MAE, and Huber loss functions. Source: Author-created. **(a)** MSE function graph; **(b)** MAE function graph; **(c)** Huber function graph.

To compare the behavior of different loss functions under small and large prediction errors, Figure 2 presents the curves of MSE, MAE, and Huber loss when the true value is fixed at zero. As is shown in Figure 2a, the mean squared error loss increases with the increase of the difference between predicted and actual value. Thus, small errors are exaggerated, and large errors induce a sharply increasing loss value. Therefore, the Mean Squared Error (MSE) is very sensitive to large errors. As shown in Figure 2b, when the predicted value matches with the actual value, the Mean Absolute Error (MAE) loss function attains minimum value of 0. In comparison with MSE, MAE grows in a stable manner as errors increase, the prediction is linearly growing without influenced by the squared errors. This shows it has much less sensitivity to outliers, thus it has higher stability when dealing with data with outliers. As shown in Figure 2c, when small errors are presented, the Huber loss function increases smoothly like MSE. When the errors are large, the growth becomes linear. Therefore, the Huber loss function balances the model’s sensitivity to outliers. The Huber loss function uses squared MAE and MSE to exhibit a medium-level of sensitivity to small errors and higher robustness to large errors.

### 2.4 Model development and reproducibility settings

To ensure reproducibility, the modeling pipeline of SSTNet was implemented as follows. First, the raw questionnaire data were screened for invalid responses, including incomplete questionnaires, duplicate submissions, and responses with obvious regular patterns. Because remaining item-level missingness after these exclusions was negligible, missing values were handled using listwise deletion. The physical education mechanism variables were treated as continuous numeric scores derived from Likert-type items; therefore, no categorical encoding was required for the predictors used in SSTNet. Continuous predictors were retained on their original Likert-derived scales, and no additional standardization was applied before tree-based training. The DASS-21 total score (dependent variable) was min–max normalized to the [0, 1] interval during training only, using the training-set minimum and maximum: y′ = (y − ymin)/(ymax − ymin).

The final dataset was randomly divided into a training set (80%, *n* = 2,983) and a test set (20%, *n* = 746). The training set was used for model fitting and hyperparameter optimization, while the test set was retained for final model evaluation. To improve the stability of model selection, 5-fold cross-validation was performed within the training set during hyperparameter optimization. The random seed was fixed at 42 for data splitting, model training, and parameter optimization.

The Sparrow Search Algorithm was used to optimize key XGBoost hyperparameters, including the number of trees (n_estimators), maximum tree depth (max_depth), learning rate (learning_rate), minimum child weight (min_child_weight), subsample ratio (subsample), column sampling ratio (colsample_bytree), L2 regularization coefficient (reg_lambda), L1 regularization coefficient (reg_alpha), and the Huber loss parameter (delta). The search ranges are shown in Table 1.

**Table 1:** Hyperparameter search ranges used in SSA optimization.

Hyperparameter	Search range
n_estimators	[100, 1,000]
max_depth	[3, 10]
learning_rate	[0.01, 0.30]
min_child_weight	[1, 10]
subsample	[0.60, 1.00]
colsample_bytree	[0.60, 1.00]
reg_lambda	[0.01, 10.00]
reg_alpha	[0.00, 5.00]
Huber delta	[0.50, 5.00]

The dependent variable was the DASS-21 total score. Following Lovibond and Lovibond, DASS-21 comprises 21 items equally distributed across depression, anxiety, and stress; item scores (0–3) are summed within each seven-item subscale and multiplied by two, and the three converted subscale scores are then summed to yield a total score with a theoretical range of 0–126 (Lovibond and Lovibond, 1995; Cao et al., 2023). Higher DASS-21 total scores indicate greater overall psychological distress, not better mental health (Lovibond and Lovibond, 1995; Cao et al., 2023). For model training, this outcome was min–max normalized to the [0, 1] interval to improve numerical stability, with ymin and ymax estimated from the training set only. After prediction, both predicted and observed values were inverse-transformed back to the original DASS-21 total-score scale before MAE and RMSE were calculated. Accordingly, all MAE and RMSE values reported in this paper are expressed in original DASS-21 total-score units (points on the 0–126 scale). R^2^ was calculated as a scale-independent goodness-of-fit indicator (Terven et al., 2025; Nagelkerke, 1991). This evaluation procedure was applied consistently to the hyperparameter-optimization, loss-function comparison, ablation, and comparative experiments.

The SSA-selected final model settings used for all subsequent experiments were n_estimators = 520, max_depth = 6, learning_rate = 0.07, min_child_weight = 3, subsample = 0.82, colsample_bytree = 0.78, reg_lambda = 1.15, reg_alpha = 0.20, and Huber delta = 1.35. All analyses were implemented in Python 3.10.12 using XGBoost 1.7.6, scikit-learn 1.3.2, NumPy 1.24.3, and pandas 2.0.3. The analysis code, random-seed configuration, and hyperparameter file are available from the corresponding author upon reasonable request.

### 2.5 Informed consent statement

The study protocol was reviewed and approved by the Institutional Review Board of Al-Farabi Kazakh National University (Approval No. AFKNU-IRB-2024-08). Authorization to conduct this study was obtained from the South China University of Technology, South China Normal University, Shenzhen University, and Foshan University. Electronic informed consent was obtained from all participants prior to data collection. Participants were provided with information sheets detailing the study’s purpose, procedures, potential risks, and data-use arrangements. The voluntary, anonymous, and confidential nature of participation was emphasized: no names or student identification numbers were collected; questionnaire responses were stored on a password-protected server accessible only to the research team; and all individuals were informed of their right to withdraw at any stage without academic or personal consequence. Consent materials were administered in Chinese, a language fully understood by the participants.

## 3 Experiments and results analysis

### 3.1 Data collection

#### 3.1.1 Participants and procedure

Participants were undergraduate students recruited from four universities in Guangdong Province, China: the South China University of Technology, South China Normal University, Shenzhen University, and Foshan University. Data were collected between September 2024 and August 2025. A multi-stage cluster sampling strategy was used. At the first stage, physical education course classes were selected as clusters within each university; at the second stage, all eligible students in the selected classes were invited to participate through physical education course instructors, university student affairs offices, an online questionnaire platform, class WeChat groups, and campus announcements.

The inclusion criteria were as follows: (1) being a registered undergraduate student at one of the participating universities, (2) having participated in university physical education courses during the survey period, and (3) being willing to provide informed consent. The exclusion criteria were (1) incomplete questionnaire responses, (2) repeated submissions, (3) response time shorter than 1 min, and (4) responses showing obvious regular patterns or inconsistent answers.

A total of 4,126 questionnaires were distributed, and 3,856 were returned (gross response rate: 93.5%). After excluding 127 invalid responses (incomplete questionnaires, *n* = 61; repeated submissions, *n* = 28; response time shorter than 1 min, *n* = 19; and patterned or inconsistent answers, *n* = 19), 3,729 valid questionnaires were retained for the final analysis, yielding a valid response rate of 90.4% (3,729/4,126). The final sample consisted of 3,729 students (1,883 men, 50.5%; 1,846 women, 49.5%), with a mean age of 21.12 years (SD = 1.31). Age statistics by academic year are reported in Section 3.1.2 and are internally consistent with the overall sample size. Participation was voluntary and anonymous. Before completing the questionnaire, all participants were informed of the purpose of the study, the confidentiality of their responses, and their right to withdraw at any time.

Gender, age, and academic year were collected to describe the demographic characteristics of the sample, but they were not included as control variables in the SSTNet model. Therefore, the modeled relationships between physical education mechanism variables and mental health outcomes should be interpreted as unadjusted predictive associations.

#### 3.1.2 Datasets and metrics

This study investigated the modeled associations between university physical education mechanisms and student mental health through a questionnaire survey, collecting 3,729 samples from students at the South China University of Technology, South China Normal University, Shenzhen University, and Foshan University. The demographic characteristics of the participants are shown in Table 2.

**Table 2:** Demographic characteristics of the participants.

Year group	Number of examples	Gender		Age / Years
		Man	Woman	
First year	964	534	430	19.81 ± 0.82
Second year	1,065	447	618	20.79 ± 0.75
Third year	1,037	578	459	21.64 ± 0.92
Fourth year	663	324	339	22.75 ± 0.87
Total	3,729	1883	1846	21.12 ± 1.31

Students’ mental health was measured using the 21-item Depression Anxiety Stress Scales (DASS-21), originally developed Lovibond and Lovibond (1995) and subsequently validated in Chinese university student samples (Cao et al., 2023). The DASS-21 contains 21 items and assesses three dimensions of psychological distress, namely depression, anxiety, and stress, with seven items for each dimension. Each item is rated on a four-point Likert scale ranging from 0 (“did not apply to me at all”) to 3 (“applied to me very much or most of the time”). Subscale scores are obtained by summing the seven items in each dimension and multiplying the score by two, yielding a theoretical range of 0–42 for each subscale, while the total score (0–126) reflects the overall level of psychological distress. Higher scores indicate higher levels of depression, anxiety, stress, or overall psychological distress; they do not indicate better mental health (Lovibond and Lovibond, 1995; Cao et al., 2023). In the predictive modeling analyses, the dependent variable was the DASS-21 total score (theoretical range: 0–126). MAE and RMSE are therefore interpretable as average absolute and root-mean-square prediction errors in original total-score points. The DASS-21 has demonstrated good reliability and validity in university student samples and has been widely used in mental health research (Cao et al., 2023). In the current study, the internal consistency was satisfactory, with Cronbach’s alpha coefficients of 0.91 for the total scale, 0.86 for depression, 0.84 for anxiety, and 0.87 for stress. This study used a cross-sectional questionnaire design. Therefore, the results should be interpreted as predictive relationships or modeled associations rather than causal effects (Savitz and Wellenius, 2023).

This study employs three metrics (MAE, RMSE, and R^2^) to evaluate the performance of the SSTNet model. Among these, MAE (mean absolute error) denotes the average absolute deviation between predicted and actual values, reflecting the average bias in model predictions (Terven et al., 2025; Willmott and Matsuura, 2005). A lower MAE indicates greater predictive accuracy. RMSE (root mean square error) is calculated by taking the square root of the average of the squared prediction errors (Terven et al., 2025; Chai and Draxler, 2014). It is more sensitive to larger errors, thus more clearly reflects significant deviations in model predictions. A smaller RMSE indicates better model performance. R^2^ (coefficient of determination) measures the model’s ability to explain variations in the data (Terven et al., 2025; Nagelkerke, 1991). Its value typically ranges from 0 to 1. The closer R^2^ is to 1, the higher the model’s fit to the data and the stronger its predictive capability. Importantly, although the outcome may be normalized during training, MAE and RMSE in all result tables are computed after inverse transformation to the original DASS-21 total-score scale (0–126).

#### 3.1.3 Physical education mechanism variables

The measurement items were adapted from previously validated instruments concerning physical activity participation, perceived exercise intensity, and teacher-student interaction quality, with wording modified to fit the university physical education classroom context (Craig et al., 2003; Torres et al., 2025). Physical education class participation frequency (PFC) was adapted from the frequency items of the International Physical Activity Questionnaire–Short Form (IPAQ-SF), which records how often respondents engage in physical activity (Craig et al., 2003). Exercise intensity adaptation (EIA) was adapted from the Borg Rating of Perceived Exertion framework and related perceived-intensity items, which are widely used to capture whether exercise load matches individual tolerance (Torres et al., 2025). Teaching interaction quality (TIQ) was adapted from the Questionnaire on Teacher Interaction (QTI), which assesses interpersonal teacher behavior and student–teacher communication in classroom settings (Khine et al., 2025). All physical education mechanism items were rated on a five-point Likert scale ranging from 1 (strongly disagree/never) to 5 (strongly agree/always). PFC was computed as a single-item frequency score, whereas EIA (4 items) and TIQ (5 items) were computed as the mean of their respective items. In the current sample, Cronbach’s alpha was 0.83 for EIA and 0.86 for TIQ. The specific survey items and scoring rules are listed in Table 3.

**Table 3:** Survey items.

Variable	Definition	Data source	Score calculation	Interpretation
PFC	Frequency of participation in university PE classes	Self-report	Single-item score	Higher scores indicate more frequent participation
EIA	Appropriateness of exercise intensity to students’ physical condition and tolerance	Self-report	Mean of 4 items	Higher scores indicate better intensity adaptation
TIQ	Perceived quality of teacher-student interaction in PE classes	Self-report	Mean of 5 items	Higher scores indicate better interaction quality

### 3.2 Ablation experiments

To comprehensively evaluate the contributions of each component within the proposed SSTNet framework, ablation experiments were conducted across three dimensions: the selection of SSA optimization strategies, the choice of loss functions, and the selection of SSTNet model architectures. Specifically, the first set of experiments validated the effectiveness of SSA in enhancing the XGBoost model by comparing its performance against other hyperparameter optimization methods such as random search and Bayesian optimization (Figure 3). The second set of experiments examined the impact of different loss functions (MSE, MAE, and Huber loss) on model prediction performance. Finally, the third set of experiments evaluated the contribution of the SSTNet architecture by comparing it with multiple baseline models. Through these experiments, the influence of each component on model accuracy and robustness can be systematically analyzed.

**Figure 3:**
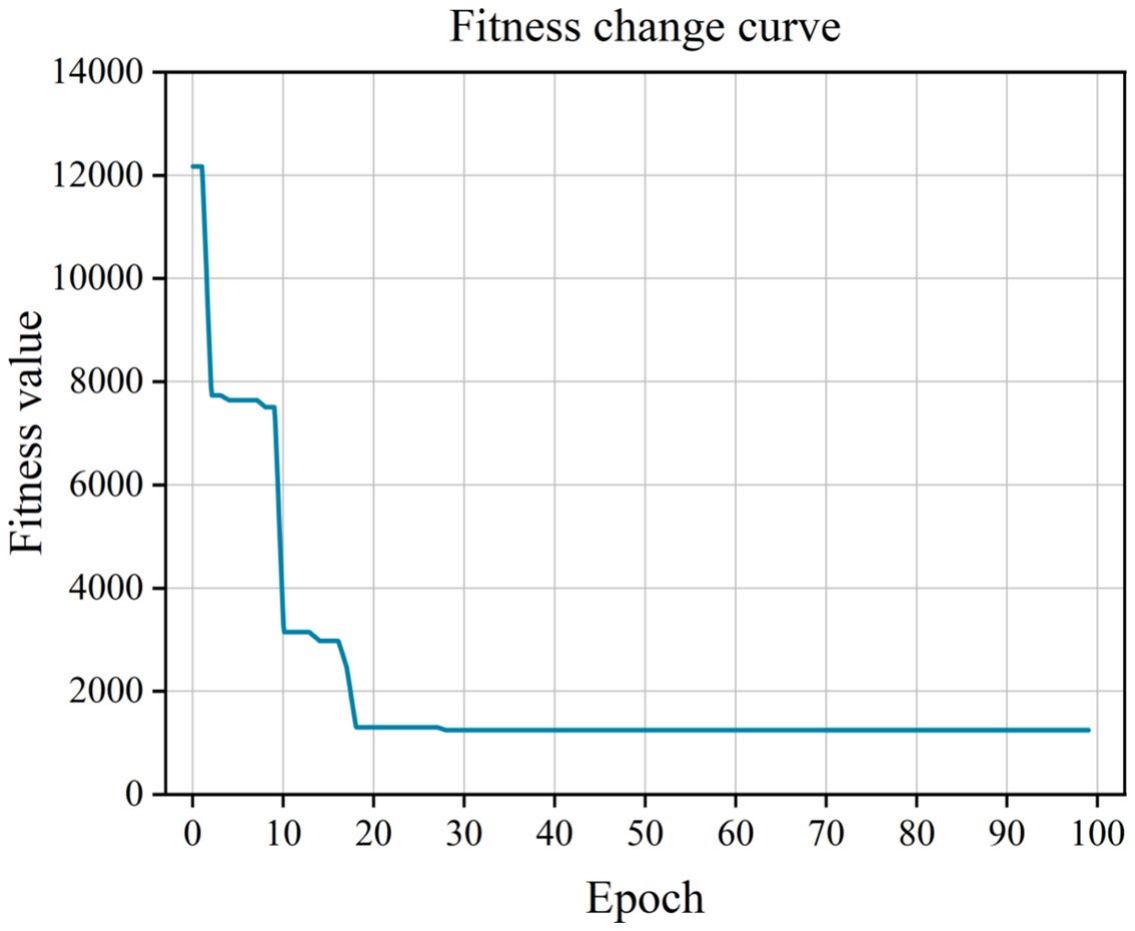
SSA algorithm optimization of XGBoost fitness value variation. Source: Author-created.

This paper employs the SSA algorithm, random search, and Bayesian optimization within the same parameter space, with the iteration count capped at 100 for each method. Subsequently, the obtained optimal parameters are applied to the SSTNet for data prediction. Model accuracy is assessed by comparing the MAE, RMSE, and R^2^ values across the three optimization approaches. The results are presented in Table 4.

**Table 4:** Prediction performance under different hyperparameter optimization strategies.

Default parameters	Random search	Bayesian optimization	SSA algorithm	MAE	RMSE	R^2^
✓				51.724	115.767	0.9389
	✓			45.632	114.984	0.9485
		✓		44.950	113.988	0.9533
			✓	40.320	108.202	0.9713

MAE and RMSE are reported on the original DASS-21 total-score scale (theoretical range: 0–126). Lower MAE/RMSE indicate better predictive accuracy; higher R^2^ indicates better model fit.

As shown in Table 4, all three optimization strategies improved the predictive performance of the model compared with the default-parameter setting. In this comparison, the default-parameter model was used as the reference model. Percentage reductions in MAE and RMSE were calculated relative to the default-parameter model, while percentage improvement in R^2^ was calculated based on the relative increase in R^2^. All MAE and RMSE values in Table 4 are reported on the original DASS-21 total-score scale (0–126).

Compared with the default-parameter model, random search reduced MAE from 51.724 to 45.632, corresponding to an 11.78% reduction, and reduced RMSE from 115.767 to 114.984, corresponding to a 0.68% reduction. The R^2^ value increased from 0.9389 to 0.9485, representing a 1.02% relative improvement.

Bayesian optimization reduced MAE from 51.724 to 44.950, corresponding to a 13.10% reduction, and reduced RMSE from 115.767 to 113.988, corresponding to a 1.54% reduction. The R^2^ value increased from 0.9389 to 0.9533, representing a 1.53% relative improvement.

SSA optimization achieved the best performance. Compared with the default-parameter model, it reduced MAE from 51.724 to 40.320, corresponding to a 22.05% reduction, and reduced RMSE from 115.767 to 108.202, corresponding to a 6.53% reduction. The R^2^ value increased from 0.9389 to 0.9713, representing a 3.45% relative improvement. These results indicate that SSA provides a more effective hyperparameter optimization strategy than random search and Bayesian optimization under the same experimental setting.

We compared the impact of integrating three loss functions (MSE, MAE, and Huber) into the combined XGBoost framework on model predictive capability. Concurrently, we calculated MAE, RMSE, and R^2^ to further analyze the predictive performance of each model. The results are detailed in Table 5.

**Table 5:** Prediction performance of XGBoost models using different loss functions.

Model	Huber	MAE	MSE	MAE	RMSE	R^2^
XGBoost			✓	44.098	110.198	0.9503
		✓		47.669	148.840	0.9256
	✓			40.320	74.941	0.9877

As shown in Table 5, the XGBoost model employing Huber loss achieved a MAE value of 40.320 and an RMSE value of 74.941 on the test set (original DASS-21 total-score scale). Both metrics were lower than those of the XGBoost models using MSE and MAE as loss functions, while its R^2^ value was the highest among the three at 0.9877. Specifically, the MAE obtained with Huber loss decreased by approximately 8.6% compared with MSE and by approximately 15.4% compared with MAE. The RMSE decreased by approximately 32.0% compared with the MSE model and by approximately 49.6% compared with the MAE model; the R^2^ value increased by approximately 3.9% compared with the MSE model and approximately 6.7% compared with the MAE model. Therefore, employing Huber loss as the loss function for the XGBoost model and utilizing the SSA algorithm to optimize both the loss function and key model parameters effectively enhances the model’s predictive accuracy and goodness-of-fit.

To evaluate the contribution of each component in SSTNet, an ablation experiment was conducted by comparing four model variants, namely SSTNet, SSA-XGBoost, Huber-XGBoost, and standard XGBoost, as shown in Table 6. The components included in each variant are defined as follows. SSTNet is the complete proposed framework, integrating SSA-based hyperparameter optimization, XGBoost modeling, and Huber loss. SSA-XGBoost combines SSA-based hyperparameter optimization with XGBoost but uses the default loss function instead of Huber loss. Huber-XGBoost combines XGBoost with Huber loss but does not use SSA-based hyperparameter optimization. Standard XGBoost serves as the baseline model and does not include either SSA-based optimization or Huber loss.

**Table 6:** Ablation experiment results.

Combination	SSA	Huber	XGBoost	MAE	RMSE	R^2^
SSTNet	✓	✓	✓	40.320	51.660	0.8300
SSA-XGBoost	✓		✓	46.620	65.520	0.7300
Huber-XGBoost		✓	✓	44.100	57.960	0.7600
XGBoost			✓	54.180	71.820	0.6800

MAE and RMSE are reported on the original DASS-21 total-score scale (theoretical range: 0–126). Lower MAE/RMSE indicate better predictive accuracy; higher R^2^ indicates better model fit.

The ablation experiment was designed to determine whether SSA-based optimization and Huber loss independently and jointly improve model performance. If SSA-XGBoost outperforms standard XGBoost, this indicates the contribution of SSA-based hyperparameter optimization. If Huber-XGBoost outperforms standard XGBoost, this indicates the contribution of Huber loss. If SSTNet outperforms both SSA-XGBoost and Huber-XGBoost, this suggests that the combination of SSA optimization and Huber loss provides additional performance benefits.

### 3.3 Comparative test

As shown in Table 7, through experimental comparison, the SSTNet model demonstrated superior predictive performance relative to GBDT, NGBoost, AdaBoost, MART, DART, Random Forest, XGBoost, LightGBM, and CatBoost across three evaluation metrics: MAE, RMSE, and R^2^.

**Table 7:** Comparative performance of different models.

Model	MAE	RMSE	R^2^
GBDT	52.920	66.780	0.6300
NGBoost	56.700	70.560	0.6800
AdaBoost	55.440	64.260	0.6900
MART	60.480	70.560	0.6200
DART	54.180	59.220	0.6700
Random Forest	54.180	61.740	0.7600
XGBoost	51.660	71.820	0.6400
LightGBM	46.620	56.700	0.7600
CatBoost	45.360	59.220	0.7900
SSTNet	41.580	50.400	0.8800

MAE and RMSE are reported on the original DASS-21 total-score scale (theoretical range: 0–126). Lower MAE/RMSE indicate better predictive accuracy; higher R^2^ indicates better model fit.

### 3.4 Evaluation of physical education mechanisms on mental health outcomes

To examine the relative contribution of physical education mechanism variables to mental health prediction, feature importance analysis was conducted using the trained SSTNet model. The initial model-based importance score was calculated from the XGBoost component of SSTNet, reflecting the average contribution of each feature to the reduction of the objective function across all decision trees. A higher score indicates that the variable contributes more to improving the model’s predictive performance.

To further verify the robustness of the feature importance results, permutation importance was calculated on the independent test set. For each predictor, its values were randomly permuted while all other variables were kept unchanged. The model performance after permutation was then compared with the original performance. A larger decrease in predictive performance indicates greater importance of the permuted variable. The permutation procedure was repeated [number] times for each variable, and the mean importance score and standard deviation were reported to provide an uncertainty estimate.

As shown in Table 8, exercise intensity adaptation (EIA) and teaching interaction quality (TIQ) had higher importance scores than physical education class participation frequency (PFC). This indicates that, within the SSTNet predictive framework, EIA and TIQ contributed more strongly to the prediction of students’ mental health outcomes than PFC. The modeled direction of association was inverse with respect to the DASS-21 total score: higher EIA and TIQ scores were associated with lower DASS-21 total scores, that is, with lower psychological distress rather than with higher distress. However, because the study used cross-sectional questionnaire data, these results should be interpreted as modeled predictive associations rather than causal effects (Savitz and Wellenius, 2023).

**Table 8:** Feature importance results of the SSTNet model.

Variable	Control conditions	Importance Score	Direction of influence
PFC	EIA and TIQ remain unchanged	0.27	Negative (higher predictor scores associated with lower DASS-21 total scores / lower distress)
EIA	PFC and TIQ remain unchanged	0.38	Negative (higher predictor scores associated with lower DASS-21 total scores / lower distress)
TIQ	EIA and PFC remain unchanged	0.35	Negative (higher predictor scores associated with lower DASS-21 total scores / lower distress)

## 4 Discussion

University students face increasing mental health risks related to academic pressure, employment uncertainty, and adaptation difficulties (Yang et al., 2025). Previous studies have reported considerable levels of depression and anxiety among Chinese university students, indicating that mental health has become an important issue in higher education (Han S. S. et al., 2025). These problems may be associated with both external pressures, such as academic competition and insufficient psychological support, and individual factors, such as emotional regulation, self-worth, and social adaptation.

Physical education may be linked to students’ mental health through physiological, psychological, and social pathways (Ren and Xiao, 2023). Moderate exercise can support emotional regulation and stress relief, while well-designed physical education programmed may help students build regular exercise habits, confidence, resilience, and social support (Zeng et al., 2025). Therefore, variables such as exercise intensity adaptation and teaching interaction quality may provide useful information for understanding students’ mental health outcomes.

To analyze these relationships more effectively, machine-learning methods have increasingly been used in mental health research (Basha et al., 2025). Compared with traditional linear approaches, machine learning can process complex and high-dimensional data, identify important predictors, and support early detection of mental health risks (de Lacy et al., 2023). This provides a methodological basis for using SSTNet to model the predictive associations between university physical education mechanisms and students’ mental health (Hale et al., 2010; Zhang L. et al., 2024).

This study developed and validated SSTNet as a robust machine-learning framework for predicting students’ mental health outcomes using university physical education mechanism variables. Based on comparative and ablation analyses, it can be observed that incorporating the Huber loss mechanism and the SSA-based parameter search strategy into the XGBoost framework leads to a notable improvement in predictive performance. Specifically, during the modeling of mental health-related data, the Huber loss reduces the influence of extreme samples and noisy observations, thereby enhancing the model’s adaptability and stability with respect to real-world educational data distributions. Meanwhile, the global hyperparameter optimization implemented by SSA effectively improves the model’s fitting quality and further strengthens its generalization capability on unseen data.

Model analysis indicates that, within the university physical education system, instructional interaction and exercise intensity are significantly associated with students’ DASS-21 total scores. A supportive teaching interaction atmosphere is associated with a stronger sense of connection and achievement and with lower reported emotional hardship. Although student engagement rates in physical activities remain relevant, their contribution should be interpreted together with course quality and intensity settings. Research findings indicate that moderate exercise intensity combined with a high-quality teaching interaction environment is associated with lower DASS-21 total scores, that is, with lower psychological distress rather than with higher distress, and with better emotional wellbeing (Zeng et al., 2025). This provides data-driven empirical evidence for university physical education curriculum reform, teaching organization optimization, and mental health promotion strategies.

This study used cross-sectional questionnaire data, which limits the ability to draw causal conclusions, a recognized limitation of cross-sectional designs. Although SSTNet can identify predictive relationships and modeled associations between physical education mechanism variables and students’ mental health outcomes, it cannot determine whether these variables causally reduce psychological distress. Recent discussions in epidemiology have reiterated that cross-sectional associations remain useful for prediction and hypothesis generation but are insufficient for causal identification. Future studies should use longitudinal, experimental, or quasi-experimental designs to examine causal mechanisms.

This study did not fully control for potential confounding variables, another long-standing concern in observational research. Although gender, age, and academic year were collected for descriptive purposes, they were not included as covariates in the SSTNet model. In addition, other potentially important factors, such as socioeconomic status, prior mental health history, academic stress, sleep quality, social support, and lifestyle behaviors, were not measured in the present survey. These unmeasured variables may be associated with both students’ physical education experiences and their mental health outcomes. Therefore, the findings should be interpreted as unadjusted predictive associations rather than causal or fully adjusted effects. Future research should include a broader set of covariates and adopt longitudinal or experimental designs to better examine the relationships between university physical education mechanisms and students’ mental health.

## 5 Conclusion

This study tested three hypotheses. H1 proposed that SSTNet would outperform baseline machine-learning models in predicting students’ mental health outcomes. The comparative results supported this hypothesis, as SSTNet achieved better predictive performance than the baseline models. H2 proposed that SSA-based hyperparameter optimization and Huber loss would improve model robustness and predictive performance. The ablation results generally supported this hypothesis by showing that the complete SSTNet model performed better than the reduced model variants. H3 proposed that exercise intensity adaptation and teaching interaction quality would be more informative predictors than physical education class participation frequency. The feature importance analysis supported this hypothesis within the present predictive model, and the modeled associations were consistent with lower rather than higher DASS-21 total scores.

Overall, SSTNet showed promising performance for analyzing noisy questionnaire-based mental health data. The findings also suggest that exercise intensity adaptation and teaching interaction quality were important predictors of students’ mental health outcomes in this dataset. However, because the study used cross-sectional self-reported data and did not fully control for potential confounders, the results should be interpreted as predictive associations with psychological distress rather than as causal effects on mental health improvement.

## Data Availability

The original contributions presented in the study are included in the article/supplementary material, further inquiries can be directed to the corresponding author.
